# Effects of Therapy at a Community Based Trauma Therapy Service Treating Child Abuse and Neglect: A Pre-Post Study Using Administrative Data

**DOI:** 10.1007/s40653-024-00625-6

**Published:** 2024-03-25

**Authors:** James Leslie Herbert, Amanda Paton

**Affiliations:** 1 Australian Centre for Child Protection, Justice & Society, University of South Australia, Adelaide, Australia; 2 Australian Centre for Child Protection, GPO Box 2471, Adelaide, South Australia, 5001 Australia

**Keywords:** Child maltreatment, Child sexual abuse, TF-CBT

## Abstract

This repeated-measures study examined the effects of a hybrid of Trauma-Focused Cognitive Behavioural Therapy (TF-CBT) with other therapeutic approaches at a community-based clinic in Perth Western Australia among a sample of children and young people overwhelmingly experiencing multiple forms of maltreatment and with complex family situations (i.e., family and domestic violence, parental mental health, parental substance abuse). Drawing on 1713 individual client records from between 2017 and 2020, the researchers identified 113 children and young people with viable pre-post treatment assessments including 78 on the TSCC, 36 on the TSCYC, and 12 on the CBCL. Significant improvements on most clinical scales were identified on the TSCC and TSCYC. Sub-analysis of the TSCC results found no differences across gender, age, care status, therapy funding source, and the presence of sexual abuse in the rate of improvement on trauma symptoms. Overall, the study highlights that integrating different therapy approaches for populations with multiple and complex trauma symptoms accessing community-based services can be useful for supporting the delivery of TF-CBT for difficult to treat populations.

## Introduction

The majority of children and young people receiving therapeutic responses for child abuse have experienced multiple forms of maltreatment (e.g., Jensen et al., 2014) which commonly manifest as a complex range of symptoms (e.g., Wolfe et al., 2006). In contrast treatments for children tend to be designed for trauma from single incidents and without complex family dynamics (Cook et al., 2005; Kliethermes et al., 2014). This study draws on data from a community-based clinic in Perth, Western Australia that provided services to children that were predominantly characterised by multiple forms of maltreatment (i.e., sexual abuse, physical abuse, emotional abuse, neglect, exposure to domestic violence) and complex presentations (i.e., a range of symptoms that may or may not be related to their maltreatment). Accordingly, this clinic adapted their therapy approach to include multiple treatment components that address the common symptoms they observed (see Chan & Herbert, 2022 for a more detailed description of this therapeutic approach). The intent of this study was to examine the effects of an existing framework of therapies implemented in a community-based setting responding primarily to multiple forms of maltreatment and complex presentations of trauma. The study sought to examine the potential benefits of this treatment approach for this population, namely the types of symptoms that appear to improve through exposure to the intervention, and whether some groups of children are more/less likely to benefit.

Across the systems that identify and respond to children affected by child abuse and neglect (i.e., community mental health, hospital, child protection) the majority of children and young people have trauma associated with multiple forms of maltreatment (e.g., Jensen et al., 2014) and symptoms that manifest in multiple and complex ways (e.g., Wolfe et al., 2006). These children and young people also tend to have ongoing complex family circumstances that can compound their trauma and complicate their treatment (Kisiel et al., 2009). In contrast, current treatments for children tend to be designed for and evidenced around responding to trauma from single incidents or series of events and without complex family dynamics that may compound the symptoms of trauma (Cook et al., 2005; Kliethermes et al., 2014). Indeed, many clinical studies routinely exclude cases with an ongoing risk of domestic violence, parental mental health, and substance abuse (Cohen & Mannarino, 2000). This highlights the difference between the populations included in clinical studies, and populations that present at community-based therapy services (Delorenzi et al., 2016).

Trauma Focused Cognitive Behaviour Therapy (TF-CBT) is a highly researched and well evidenced intervention for children and young people with trauma from abuse and neglect, which is routinely found to outperform client-focused support treatment conditions in symptom reduction (e.g., Cohen & Mannarino, 1996, 2000). However, much of the evidence for TF-CBT comes from randomised trials that set exclusion criteria that limit the complexity of cases (e.g., exclusions for parental mental health issues, substance abuse, ongoing family and domestic violence). TF-CBT is oriented towards responding to child sexual abuse in isolation from a complex context (e.g., Self-Brown et al., 2016; Celano et al., 2018), with this reflected in the format of the program involving identifying and processing the single most intense trauma (Cohen & Mannarino, 1996). Recognised limitations with TF-CBT in relation to treating Complex Trauma have resulted in the adaption of the treatment protocol to respond to both more intense symptoms and the more complex circumstances of these cases (Cohen et al., 2012).

The recognition of Complex Post-Traumatic Stress Disorder (CPTSD) in diagnostic classifications (e.g., World Health Organization, 2018) has highlighted the potential challenges in treating these disorders, and questions about the applicability of the evidence base for well-established Post-Traumatic Stress Disorder (PTSD) treatments such as TF-CBT (e.g., Maercker et al., 2022). This has prompted adaptations to the existing treatment for PTSD such as extending the total length of treatment and altering the length and intensity of different components within the treatment (Cohen et al., 2012). Consistent with this, researchers running TF-CBT treatment trials with this population have observed the need for a more individualised approach to treatment, building off TF-CBT as a base, but adapting the approach and including additional modules to address additional symptoms (e.g., dysregulation, relational and social difficulties; Hébert & Amédée, 2020). Current expert developed guidelines suggest that the choice, combination and sequence of treatments need to be tailored based on the symptoms and needs of the patient (Bisson et al., 2019).

### Treatment Adaption in the Current Study

The current study reports on the treatment effects of a service in Perth, Western Australia delivered by Parkerville Children and Youth Care Inc. known as the Therapeutic Family Service. The service primarily provided TF-CBT (Chan & Herbert, 2022) but compared to the standard treatment model it was typically over a longer period of time and often with many more sessions (Cohen & Mannarino, 1996), even compared to the recommended adaption of TF-CBT for Complex Trauma (Cohen et al., 2012). The practice framework for the service also involved several additional components of treatments to address symptoms identified during the assessment process. In a separate qualitative study of the treatment model (see Chan & Herbert, 2022) clinicians indicated these additional components most commonly included Eye Movement Desensitisation and Reprocessing, Acceptance and Commitment Therapy, Imagery Rescripting, Dyadic Developmental Psychology, Dialectical Behavioural Therapy, Schema Therapy, and others. The matching of additional components to symptoms were determined by the Parkerville clinical team and the Director of Therapeutic & Advocacy Services based on the evidence for the types of treatments associated with addressing individual psychological symptoms. The treatment length was variable and was often affected by the type of program or referral source, with referrals from the multi-agency response and the community-based program having 26 sessions, referrals from the state statutory child protection agency often including services longer than a year, whereas self-referrals typically only included 10 sessions per year under a mental health care plan through Medicare, unless families elected to self-fund additional sessions.

The service responded a range of different categories of children that could potentially experience different effects from the treatment^1^. In a similar sample Ascienzo et al. (2022) found differences in symptom improvement between male and female poly-traumatised children at different phases of treatment, noting higher scores on some symptoms for females at baseline, PRAC^2^ skills, and trauma narrative phases, but no difference overall. Given the rates of mid-treatment dropout among referred samples (Herbert, 2021), there is the potential for females to have a higher level of symptoms if the treatment ceased at these earlier phases. Researchers have highlighted less consistent outcomes across studies delivering TF-CBT for younger children (e.g., McGuire et al., 2021), highlighting that some elements of the model assume developmental milestones that younger children may not have reached. Symptom improvement could potentially vary between different referral streams in TFS, particularly as the service included a priority response from a co-located multi-agency investigation team (Herbert & Bromfield, 2020) likely to refer children that had just disclosed, as well as self-referrals and referrals from the state child protection agency. The sample also included children in Out of Home Care, meaning elements of TF-CBT oriented to caregivers had to be adapted for carers (Chan & Herbert, 2022), and be responsive to likely a different scale of trauma and attachment issues. The presence of sexual abuse in the history of children is associated with a different symptom profile (e.g., Stanaway et al., 2018), although as noted it is common in this type of sample to have multiple forms of maltreatment (Jensen et al., 2014).

This study will examine the treatment effects of this therapy framework and examine how different characteristics of trauma symptoms respond to this treatment, and whether different client groups benefited disproportionately from the approach. The research questions include:

1. Does the treatment reduce the symptoms of trauma among a community sample of children and young people who have typically experienced multiple maltreatments and complex presentations of trauma?

2. Are different client characteristics associated with different rates of trauma symptom improvement?

## Methods

This study undertook a retrospective pre-post analysis to assess the treatment effects of a community-based therapy oriented towards multiple maltreatment and complex presentations of trauma. The researchers obtained de-identified administrative data from the agency and cases were identified/selected based on valid pre-post applications of the same psychometric instrument to examine the effects of this therapy in a community treatment setting. The study represents an uncontrolled repeated measures design, which has the advantage of more closely representing realistic conditions of therapy but has the limitation of non-standardised assessment schedules and a non-randomised sample. These limitations have been controlled by screening for inclusion and reporting other variations that may have affected the treatment effect (e.g., number of therapy sessions prior to the pre-treatment assessment). For this study a valid pre-post required the instruments to be administered over the same discrete course of treatment (> 6 sessions), as many of the clients had multiple courses of treatment with the same provider. Six sessions was chosen as the minimum number of sessions for a valid treatment as clinicians in the service identified as the number of sessions where a therapist could feasibly work through the minimum required content of the therapy, noting that sessions typically involved a number of rapport building sessions before administering the psychometric instruments (Chan & Herbert, 2022).

### Sample

Therapeutic Family Services (TFS) was a directorate within Parkerville that provided psychology services in a community setting in Perth, Western Australia, along with some regional services in the South-West and Wheatbelt regions of Western Australia. The service responded almost exclusively to cases with multiple forms of maltreatment and complex symptoms and circumstances, receiving referrals from a co-located investigative response for child sexual abuse, the organisation’s out-of-home care, funded therapy placements from the state statutory child protection agency, a community-based program, and some Medicare and self-funded clients.

This study sought data from the treatment service inclusive of all cases seen between 2017 and 2020 that had an administration of a psychometric instrument on their case record, excluding ongoing cases at the time of the data request (October 2020). While 1713 individual children/young people were seen in total over the relevant period, the service often did assessments for other programs and did not provide treatment themselves, and many cases only had a single psychometric instrument in the case record completed typically at the time of assessment.

**Fig. 1:**
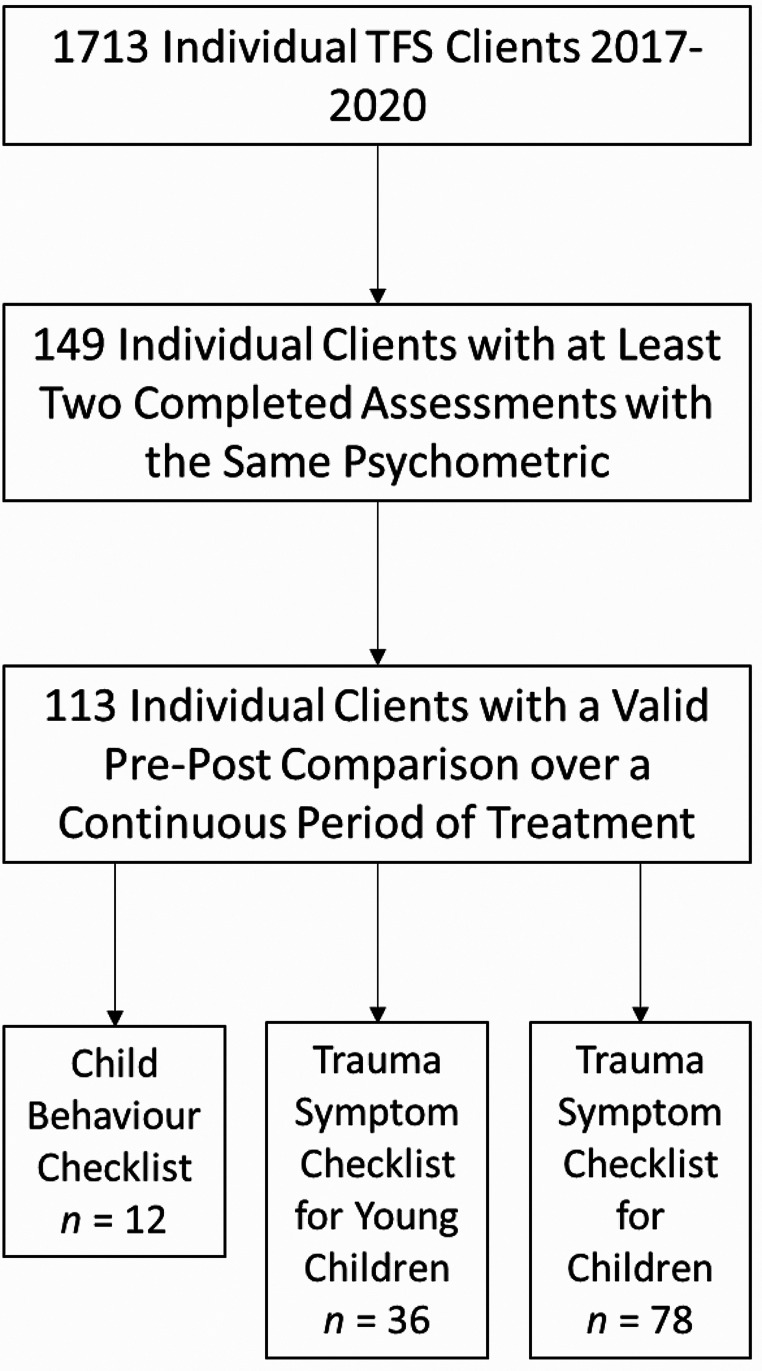
Diagram of Sample Selection

Of the 149 eligible cases extracted (i.e., relevant treatment by Parkerville and at least two administrations of a psychometric instrument), only 113 were found to have a valid pre-post on the same psychometric instrument and have a valid length of treatment (> 6 sessions). This threshold of six sessions was recommended by the clinical team at Parkerville to help distinguish between cases that may have been attending sessions just to undergo assessment as part of being referred to another service. The clinical team also identified that six sessions as part of a discrete block of treatment was the minimum number of sessions to work through enough of the therapy content to plausibly see some improvement in symptoms; noting that usually 2–3 rapport building sessions occurred before any psychometric instruments were completed. All cases were within the expected age range for the program (< 18 years old). To determine validity the treatment sessions were mapped against the administrations of each of the psychometric instruments to identify whether the pre-post could be mapped to a discrete period of treatment, as many of the cases involved multiple engagements and dis-engagements with therapy. This also resulted in the screening out of some cases where the relevant treatment period for the observation was too short (< 6 sessions).

The final sample was compared against the treatment population the data was drawn from (see Table 1), which identified some significant differences. The analysis found that while the sample was equivalent to the treatment population on most characteristics, the sample had a significantly larger proportion of cases in the care of the CEO (χ^2^ (1, *n* = 1677) = 12.98, *p* = < 0.004*), cases with parental mental health flagged as a concern (χ^2^ (1, *n* = 1090) = 22.79, *p* = < 0.004*), and cases with sexual abuse as a concern (χ^2^ (1, *n* = 1090) = 9.04, *p* = < 0.004*). This reflects that cases with these characteristics were more likely to receive multiple psychometric instruments, especially children in care who were required to have these measures as part of their case reports. Similarly, children with higher complexity were likely to have higher pre-treatment assessments inclusive of psychometric instruments which would have increased the likelihood of a similar follow-up assessment towards the end of treatment. While it is preferrable methodologically that a sample is representative of the population, these differences were not unexpected based on the need for the closer monitoring of the symptoms of children in out-of-home care, in complex home situations (i.e., parental mental health concerns), and children with histories of sexual abuse.

**Table 1:** Comparison of Treatment Population with Sample

	Population (*n* = 1564)	Sample (*n* = 113)	Sig Testing^1^
Sex^2^			χ2 (1, *n* = 1626) = 3.50, *p* = .06^3^
*Female*	764 (50.2%)	65 (57.5%)	
*Male*	753 (49.5%)	44 (38.9%)	
*Diverse Gender Identity*	4 (0.3%)	1 (0.9%)	
Age	10.34 (3.34)	10.84 (2.86)	*U* = 79927.500, *z* = -1.70, *p* = .088
Care Status (in care)	565 (36.1%)	60 (53.1%)	χ2 (1, *n* = 1677) = 12.98, *p* = < 0.004*
Primary Concern^4^			
*Neglect*	184 (23.7%)	20 (21.3%)	
*Sexual Abuse*	239 (30.8%)	39 (41.5%)	
*Physical Abuse*	76 (9.8%)	8 (8.5%)	
*Witness Domestic Violence*	165 (21.3%)	17 (18.1%)	χ2 (4, *n* = 748) = 3.50, *p* = .321
Parental Drug and Alcohol^5^	393 (40.1%)	56 (49.6%)	χ2 (1, *n* = 1090) = 4.77, *p* = .029
Parental Mental Health^5^	231 (23.6%)	49 (55.5%)	χ2 (1, *n* = 1090) = 22.79, *p* = < 0.004*
Parental Capacity^5^	413 (42.1%)	61 (55.5%)	χ2 (1, *n* = 1090) = 12.98, *p* = .008
Physical Abuse^5^	262 (26.7%)	28 (25.5%)	χ2 (1, *n* = 1090) = 0.08, *p* = .773
Sexual Abuse^5^	267 (27.2%)	45 (40.9%)	χ2 (1, *n* = 1090) = 9.04, *p* = < 0.004*
Emotional Abuse^5^	270 (27.6%)	41 (37.3%)	χ2 (1, *n* = 1090) = 0.4.58, *p* = .032
Experience of Neglect^5^	376 (38.4%)	50 (45.5%)	χ2 (1, *n* = 1090) = 2.09, *p* = .149
Witnessing Domestic Violence^5^	477 (48.7%)	54 (49.1%)	χ2 (1, *n* = 1090) = 0.007, *p* = .934
Living Situation at Intake			χ2 (3, *n* = 1534) = 12.01, *p* = .007
*OOHC*	363 (25.5%)	43 (38.4%)	
*With Parents*	695 (48.7%)	38 (33.9%)	
*Extended Relatives*	264 (18.5%)	18 (16.1%)	
*Alternate Parent*	104 (7.3%)	9 (8.0%)	

^1^<0.05 with a Bonferroni Correction to 0.004

^2^For 51 cases the gender identity was missing from the case record

^3^Note: Due to small numbers of ‘diverse gender identity’ this analysis was restricted to proportions of female and male

^4^Note: For 929 cases the primary concern was missing from the case record

^5^Note: For 587 cases the presence/absence of concerns were missing from the case record

### Treatment

The treatment provided by Parkerville’s specialist psychology service TFS was primarily based on TF-CBT, with most clinicians indicating this was the approach they aligned most closely to (Chan & Herbert, 2022). This multi-modal approach was implemented in recognition of the complex backgrounds and multiple forms of maltreatment experienced by most of the children and young people attending the clinic, consistent with the most up to date understandings of child abuse and neglect (Higgins et al., 2023). The most common therapeutic approaches delivered in the program included TF-CBT, EMDR, Cognitive Behavioural Therapy, Acceptance and Commitment Therapy, Imagery Rescripting, Dyadic Development Psychology, Dialectical Behaviour Therapy, Scheme Therapy, and Circle of Security (Chan & Herbert, 2022). The treatment approach was determined through formulation in group supervision with the clinical leads and clinical director, applying an informal matrix of treatments matched to symptoms. In a separate study staff noted that the ‘Bruce Perry Neuro-sequential Model of Therapeutics’ was the model for how they structured their multi-modal treatment approach.

### Instruments

The TFS undertook a variety of assessments with clients, although the choice of assessments was up to the individual discretion of the clinician. While intended that psychometric instruments were used at the beginning and ending of treatments, the point at which instruments were used was ultimately determined by the clinicians with their clients; in many cases initial assessments were not completed until the clinician had the opportunity to establish a therapeutic alliance with the client. Similarly, some children suddenly disengaged from therapy making it not possible to complete an end of treatment assessment.

The suite of assessment tools used by TFS are common psychological instruments used for measuring the symptoms of trauma. The administrative data included: The Child Behaviour Checklist (CBCL; *n* = 28) and accompanying Youth Self Report (YSR; *n* = 3), the Trauma Symptom Checklist for Children (TSCC; *n* = 97) or Trauma Symptoms Checklist for Young Children (TSCYC; *n* = 48), the Beck Youth Inventory (*n* = 7), Child Revised Impact of Event Scale (CRIES; *n* = 15), Adolescent Dissociative Events Scale (A-DES; *n* = 1), Depression Anxiety Stress Scales (DASS; *n* = 7), and the CBCL 1.5 (*n* = 1). As many of these instruments had a small number of pre-post administrations, the analysis was restricted to the TSCC and the TSCYC.

### TSCC & TSYCC

The TSCC and TSYCC are standardised trauma measures assessing acute and chronic post-traumatic stress and other psychological symptoms associated with trauma. The TSCC is a 54 item self-report instrument for children ages 8–16 years who have experienced a traumatic event (Briere, 1996). TFS clinicians sometimes also used the TSCC-A (44 items), which is the same instrument but without items covering sexual content. Children are asked to indicate whether each of the items occur (0 = never, 1 = sometimes, 2 = lots of times, 3 = almost all of the time) over the last month, across six clinical scales including: Anxiety, Depression, Post-Traumatic Stress, Sexual Concerns, Dissociation, and Anger. Briere (1996) reported Cronbach’s alpha coefficients ranging from 0.77 to 0.89 for the clinical scales and 0.84 for the complete scale. The TSYCC is an adaption of this instrument for use with younger children (3–12 years) by their caregiver (Briere, 2005). It includes 90 items reporting on eight clinical scales: Anxiety, Depression, Anger/Aggression, Posttraumatic Stress - Intrusion, Posttraumatic Stress - Avoidance, Posttraumatic Stress - Arousal, Dissociation, and Sexual Concerns.

### Client Demographic Data

In addition to the data relating to the pre and post treatment assessments the following client information was collected:

• Client age at intake and at each administration of a psychometric instrument.

• If the child is in out-of-home care.

• Client sex.

• Client ethnicity.

• Referral source and funding for therapy.

• Date of referral, commencement, and end of therapy.

• Living situation at intake and conclusion of therapy.

• Primary abuse type reason for the referral (i.e., witness FDV, physical abuse, sexual abuse) and the presence of other forms of adverse childhood experiences including other forms of abuse in case history (i.e., neglect, parental poor mental health, parental drug and alcohol abuse).

• Number of therapy sessions and dates of therapy sessions.

### Procedures

Data was extracted by staff from Parkerville Children and Youth Care Inc. and a data analyst contracted by Parkerville and provided to the research team as part of an administrative data request. Primarily this involved fixed field information from the Parkerville administrative data system being exported into an excel sheet, or information recorded in the database in pdf form being manually copied by coders into the data sheet.

While each of the client fields were populated with information generated by the Parkerville database, the assessment results for each instrument were attached to the database as a pdf with results handwritten in some cases (e.g., with the TSCC and TSCYC). Extraction of results involved two Parkerville staff entering the results of these tests into an excel spreadsheet. These staff undertook 25% double entry across each of the cases to determine the rate at which data entry errors occurred; no discrepancies were identified among the double coded cases.

Client demographics were obtained for all cases with at least two administrations of the same psychometric instrument. Dates in the demographics were used to generate the following variables: age at first assessment, number of days from referral to commencement of therapy; number of days from commencement to discharge; days between pre and post assessments. As each case included all interactions with the client, these were limited to interactions that counted as in-person therapy sessions (i.e., ‘assessment’, ‘individual contact’, ‘safety/risk assessment’, ‘couple/family contact’). Limited to the sessions that the client attended, this information was used to generate the number of attended sessions between pre and post, and the number of sessions that occurred before the pre assessment. Particularly for more complex cases, clinicians often spent several sessions building rapport and working to stabilise the child and family before administering the first psychometric instrument.

Several procedures were undertaken to screen the data for eligible pre and post assessments. As many of the children had multiple treatment periods, treatment engagements were mapped to help visualise the relationship between the treatment periods and when the assessments occurred. 60 days between sessions was used as the threshold to separate discrete periods of treatment. This helped to highlight where assessments did not reflect pre-post treatment, many of which reflected multiple pre-treatment assessments occurring at the beginning of different treatment periods. Individual tests were also ruled ineligible for the pre-post where less than 6 therapy sessions occurred between the pre-post. This led to 17 CBCL, 19 TSCC, and 12 TSCYC administrations being identified as ineligible for the pre-post comparison.

### Ethics

The study was approved by the University of South Australia’s Human Research Ethics Committee, with organizational approvals from Parkerville’s Senior Leadership Group including the Chief Executive and Directors. The study managed potential ethical risks by obtaining only de-identified data, with cases identified by codes that only Parkerville staff could match back to identifying details. All clients receiving services at Parkerville signed a service agreement noting that their data may be used for research or program review. As the participants were children and young people this service agreement was signed by the caregiver/carer and serves as assent for inclusion in the research. While the data obtained were sensitive, the risks to children and families were low due to the use of de-identified data. As a small proportion of the sample were Aboriginal or were from culturally and linguistically diverse communities, too small for meaningful analysis, the paper does not report separate results by race/ethnicity.

### Analysis

For both the TSCC and the TSCYC a repeated samples t-test (or an Wilcoxon Signed-Rank Test where the samples were not normally distributed) was used to examine whether a significant difference existed between the pre and post treatment measures. Changes in clinical significance were examined using the McNamar’s test, and effect sizes were calculated using Cohen’s D. For the TSCC, an independent samples t-test (or an Independent Samples Mann-Whitney U where the samples were not normally distributed) was conducted, using differences between pre and post-tests as the dependent variable, and gender, care status, therapy funding, presence of child sexual abuse, living situation at intake and age categories as independent variables. A Bonferroni adjusted significance level was used to correct for multiple comparisons within each of the scales within the included instruments.

A power analysis conducted with G*Power indicated that the required samples for a repeated samples t-test was 34 to achieve a power of 80% and a level of significance of 5% (two sided), for detecting an effect size of 0.5 between pairs. This means that the comparison of means for the CBCL scales was under powered, while the comparisons for the TSCYC and TSCC were adequately powered. This informed the decision to not include the analysis of results from the CBCL in this paper. For an independent samples t-test the required sample was 64 to achieve a power of 80% and a level of significance of 5% (two sided), for detecting an effect size of 0.5 between groups. This meant that the analyses of how demographic factors influenced the mean differences between pre-post on the TSCC was adequately powered.

## Results

Two psychometric instruments (TSCYC and TSCC) were included in the analysis of differences between pre-treatment and post-treatment. Some additional analyses were conducted with the TSCC results as the larger sample size allowed for a comparison of how different demographic factors influenced treatment effects.

As relevant treatment periods were linked to when the eligible pre-post occurred, treatment characteristics are separated by individual instruments (see Table 2); because of the small number of eligible comparisons, the treatment effects from the CBCL have not been reported. As reports were by treatment period and instrument this meant that in some circumstances results could include the same client more than once for the same or even different periods of treatment if an eligible pre-post occurred with more than one instrument. For the TSCC there were an average of 229 days between pre and post tests, with an average of 16 attended sessions between observations. On average there were around 4 attended sessions prior to the pre-test. Results were similar for the TSCYC on days between pre and post-tests (*m* = 241.9; *sd* = 126.2), number of attended sessions between observations (*m* = 16.0; *sd* = 8.9), and number of attended sessions prior to the pre-test (*m* = 2.9; *sd* = 4.1).

Across all eligible cases (*n* = 113) the average time a case was active with the treatment provider was just over a year (*m* = 422 days: *sd* = 214 days), although this was based on the length of time a case was open; cases could remain open a long time without activity. There was on average 114 days (*sd* = 165) between the date of referral and the commencement of therapy. The clinician rated reason for discharge was most commonly ‘normal completion’ (*n* = 55; 48.7%), ‘discharged’ (*n* = 29; 25.7%), with a smaller number of cases having ‘funding ceased’ (*n* = 3; 2.7%), ‘mutual agreement to discharge early’ (*n* = 8; 7.1%), ‘withdrawal prior to completion’ (*n* = 3; 2.7%), and ‘other’ (*n* = 11; 9.7%).

**Table 2:** Treatment Characteristics

	CBCL (*n* = 12)	TSCYC (*n* = 36)	TSCC (*n* = 78)	Sample (*n =* 113)
Days between Pre-Post	200.3 (105.9)	241.9 (126.2)	229.0 (135.7)	
Number of Attended Sessions between Pre-Post	12.5 (4.8)	16.3 (8.8)	16.1 (8.8)	
Number of Attended Sessions Prior to Pre-Test	4.6 (6.3)	3.0 (4.1)	3.6 (5.1)	
Days the Case was Active with the Treatment Provider	354.3 (221.6)	435.1 (218.8)	433.2 (212.3)	422.1 (214.4)
Days Between Referral and First Therapy Session	123.0 (160.02)	103.5 (131.6)	123.2 (185.3)	114.6 (165.2)

### TSCYC Results

Significant improvements were identified between pre and post treatment on TSCYC anxiety (*Z* = -3.38, *p* = .001, *d* = 0.46), depression (*t* = 3.40, *p* = .002, *d* = 0.57), anger/aggression (*t*(35) = 4.21, p =.<001, *d* = 0.70), post-traumatic stress – intrusion (Z = -3.84, p = < 0.001, *d* = 0.72), post-traumatic stress – arousal (*t* = 3.94, p = < 0.001, *d* = 0.62), and post-traumatic stress – total (*t* = 5.08, p = < 0.001, *d* = 0.67) scales (see Table 3). All effect sizes were between the ‘medium’ to ‘large’ range (Cohen, 1988). No significant differences were identified for post-traumatic stress - avoidance (t = 2.29, *p* = .028), dissociation (t = 1.96, *p* = .057), and sexual concerns (*Z* = -1.32, *p* = .187). In terms of changes to the presence of clinically significant symptomatology, significant differences were identified for anxiety (0.002), anger/aggression (0.001), post-traumatic stress – intrusion (0.002), post-traumatic stress – arousal (0.001), and post-traumatic stress – total (< 0.001). Among all scales with a significant difference in terms of change on symptom scales, only depression did not also have a significant difference in terms of changes to clinical symptom status.

**Table 3:** TSCYC Scores Pre and Post Treatment (*n* = 36)

	Pre-Treatment		Post-Treatment			
	Score *m* (sd)	Clinical Sig^1^	Score *m (sd)*	Clinical Sig	Sig Testing^2^	Sig Testing (Clinical Sig)^3^
*Clinical Scales*						
Anxiety	68.61 (18.10)	C = 17 B = 6 *N* = 13	57.94 (12.48)	C = 6 B = 5 *N* = 25	*Z* = -3.38, *p* = .001, *d* = .46^4^*	0.002*
Depression	67.31 (17.55)	C = 18 B = 4 *N* = 14	57.86 (12.53)	C = 7 B = 7 *N* = 22	*t*(35) = 3.40, *p* = .002, *d* = 0.57*	0.035
Anger/ Aggression	75.17 (19.08)	C = 23 B = 5 *N* = 8	63.58 (16.13)	C = 9 B = 4 *N* = 23	*t*(35) = 4.21, *p* =.<001, *d* = 0.70*	0.001*
*Post-Traumatic Stress*						
Post-Traumatic Stress – Intrusion	67.47 (17.73)	C = 14 B = 5 *N* = 17	56.83 (12.99)	C = 5 B = 4 *N* = 27	*Z* = -3.84, *p* = < 0.001, *d* = 0.72*	0.002*
Post-Traumatic Stress - Avoidance	71.08 (21.48)	C = 16 B = 4 *N* = 16	63.67 (19.92)	C = 10 B = 3 *N* = 23	*t*(35) = 2.29, *p* = .028, *d* = 0.38	0.092
Post-Traumatic Stress - Arousal	69.25 (15.00)	C = 15 B = 9 *N* = 12	61.08 (13.21)	C = 9 B = 3 *N* = 24	*t*(35) = 3.94, *p* = < 0.001, *d* = 0.62*	0.001*
Post-Traumatic Stress - Total	73.17 (16.72)	C = 19 B = 5 *N* = 12	62.08 (15.24)	C = 7 B = 6 *N* = 23	*t*(35) = 5.08, *p* = < 0.001, *d* = 0.67*	< 0.001*
*Dissociation/ Sexual*						
Dissociation	64.19 (15.78)	C = 12 B = 4 *N* = 20	59.97 (16.58)	C = 8 B = 4 *N* = 24	*t*(35) = 1.96, *p* = .057, *d* = 0.33	0.549
Sexual Concerns	64.37 (20.19)	C = 11 B = 9 *N* = 15	59.54 (18.54)	C = 7 B = 3 *N* = 25	*Z* = -1.32, *p* = .187, *d* = 0.21	0.057

^1^C = Meets the threshold for clinical significance; B = Borderline for clinical significance; N = Below threshold for clinical significance

^2^<0.05 with a Bonferroni Adjusted Alpha to 0.006

^3^Comparisons of clinical significance were made using McNemar’s test, with borderline results combined with clinically significant results

^4^Cohen’s d from https://memory.psych.mun.ca/models/stats/effect_size.shtml

### TSCC Results

Significant differences were identified on anxiety (*t* = 5.73, p = < 0.001, *d* = 0.64), depression (*t* = 5.52, p = < 0.001, *d* = 0.62), anger (*Z* = -4.90, p = < 0.001, *d* = 0.57), post-traumatic stress (*t* = 7.17, p = < 0.001, *d* = 0.81), dissociation (*t* = 3.96, p = < 0.001, *d* = 0.45), dissociation – overt (*t* = 3.97, *p* = .001, *d* = 0.45), and sexual concerns – distress scales (*Z* = -2.78, *p* = .005, *d* = 0.38) of the TSCC (see Table 4). Effect sizes were mostly between the ‘medium’ and ‘large’ range, although improvement on the post-traumatic stress scale indicated a fairly large effect size (*d* = 0.81). No significant differences were identified for anger (*t* = 2.10, *p* = .039), dissociation – fantasy (*t* = 2.10, *p* = .039), sexual concerns (*t* = 2.74, *p* = .009), sexual concerns – pre-occupation (*Z* = -2.03, *p* = .042). In terms of the proportions with clinically significant symptoms anxiety (< 0.001), depression (0.002), post-traumatic stress (< 0.001) scales all had significant differences. Anger, dissociation – overt, sexual concerns – distress all had a significant change in symptoms but not a significant change in the proportions with clinically significant symptoms.

**Table 4:** TSCC Scores Pre and Post Treatment (*n* = 78)

	Pre-Treatment		Post-Treatment			
	Score *m* (sd)	Clinical Sig^1^	Score *m* (sd)	Clinical Sig	Sig Testing^2^	Sig Testing^3^ (Clinical Sig)
*Scales*						
Anxiety	58.24 (13.97)	C = 27 B = 27 *N* = 24	50.00 (11.12)	C = 8 B = 31 *N* = 39	*t*(77) = 5.69, *p* = < 0.001, *d* = .64^4^*	< 0.001*
Depression	56.18 (11.47)	C = 18 B = 38 *N* = 22	48.83 (9.80)	C = 8 B = 25 *N* = 45	*t*(77) = 5.45, *p* = < 0.001, *d* = 0.62*	0.002*
Anger	53.90 (11.22)	C = 14 B = 37 *N* = 27	47.45 (8.87)	C = 3 B = 31 *N* = 44	*Z* = -4.86, *p* = < 0.001, *d* = 0.58*	0.007
Post-Traumatic Stress	57.54 (11.26)	C = 21 B = 38 *N* = 19	48.28 (9.24)	C = 5 B = 28 *N* = 45	*t*(77) = 7.09, *p* = < 0.001, *d* = 0.80*	< 0.001*
*Dissociation*						
Dissociation	57.04 (11.86)	C = 20 B = 37 *N* = 21	52.13 (10.46)	C = 10 B = 31 *N* = 37	*t*(77) = 3.87, *p* = < 0.001, *d* = 0.44*	0.021
Dissociation – Overt	58.05 (12.29)	C = 24 B = 34 *N* = 20	52.99 (10.75)	C = 9 B = 36 *N* = 33	*t*(77) = 3.87, *p* = .001, *d* = 0.44*	0.007
Dissociation – Fantasy	53.06 (10.93)	C = 12 B = 36 *N* = 30	50.63 (9.60)	C = 6 B = 33 *N* = 39	*t*(77) = 2.05, *p* = .039, *d* = 0.23	0.019
*Sexual*						
Sexual Concerns	57.24 (18.03)	C = 12 B = 14 *N* = 27	52.28 (16.70)	C = 7 B = 17 *N* = 27	*t*(45) = 2.61, *p* = .009, *d* = 0.38	0.039
Sexual Concerns – Pre-Occupation	52.58 (15.98)	C = 4 B = 17 *N* = 31	50.20 (14.71)	C = 6 B = 13 *N* = 32	*Z* = -1.92, *p* = .055, *d* = 0.21	1.00
Sexual Concerns - Distress	63.52 (22.56)	C = 16 B = 15 *N* = 19	57.15 (18.90)	C = 11 B = 15 *N* = 25	*Z* = -2.72, *p* = .007, *d* = 0.37*	0.125

^1^C = Meets the threshold for clinical significance; B = Borderline for clinical significance; N = Below threshold for clinical significance

^2^<0.05 with a Bonferroni Adjusted Alpha to 0.005

^3^Comparisons of clinical significance were made using McNemar’s test, with borderline results combined with clinically significant results

^4^Cohen’s d from https://memory.psych.mun.ca/models/stats/effect_size.shtml

### TSCC Comparison with Demographic Characteristics

The TSCC had a large enough sample to allow for some comparisons of how demographic factors may have influenced improvements on trauma symptoms. These were examined using the difference between pre-post scores as the dependent variable.

### Sex

Comparing males and females on differences between pre-post on each of the TSCC scales, no significant differences were identified (see Table 5). Males (*m* = 0.35; *sd* = 9.25) on average did not improve as much on the Dissociation – Fantasy scale compared to females (*m* = 3.64; *sd* = 11.19), however this difference did not reach significance.

**Table 5:** Comparison of Symptom Change between Sex (*n* = 75)

	Male Score m (sd)	Female Score m (sd)	Sig Testing^1^
*Scales*			
Anxiety	(*n* = 26) 8.88 (14.37)	(*n* = 49) 7.82 (12.41)	*U* = 617.000, *p* = 8.22
Depression	(*n* = 26) 8.12 (12.31)	(*n* = 49) 7.14 (12.13)	*U* = 634.000, *p* = .973
Anger	(*n* = 26) 8.31 (14.66)	(*n* = 49) 5.53 (9.11)	*U* = 565.500, *p* = .426
Post-Traumatic Stress	(*n* = 26) 10.62 (11.84)	(*n* = 49) 8.61 (11.66)	*U* = 588.000, *p* = .585
*Dissociation*			
Dissociation	(*n* = 26) 4.31 (12.29)	(*n* = 49) 5.12 (10.99)	*U* = 618.500, *p* = .837
Dissociation – Overt	(*n* = 26) 4.92 (13.34)	(*n* = 49) 4.92 (10.81)	*U* = 607.500, *p* = .793
Dissociation – Fantasy	(*n* = 26) 0.35 (9.25)	(*n* = 49) 3.61 (11.31)	*U* = 508.500, *p* = .151
*Sexual*			
Sexual Concerns	(*n* = 9) 12.33 (9.80)	(*n* = 35) 3.37 (13.35)	*U* = 91.000, *p* = .051
Sexual Concerns – Pre-Occupation	(*n* = 8) 7.12 (10.58)	(*n* = 35) 1.57 (11.91)	*U* = 117.500, *p* = .490
Sexual Concerns - Distress	(*n* = 9) 13.22 (19.08)	(*n* = 35) 4.91 (16.82)	*U* = 127.500, *p* = .389

^1^ <0.05 with a Bonferroni Adjusted Alpha to 0.005

### Age

No significant differences were identified between age groups (7–12 & 13–17) on improvements on any scales on the TSCC (see Table 6), although older children on average did not improve on the Sexual Concerns Pre-Occupation scale (*m* = − 0.46; *sd* = 12.78), while younger children showed a minor improvement (*m* = 5.62; *sd* = 9.22).

**Table 6:** Comparison of Symptom Change between Age Groups (*n* = 78)

	7-12-Year-Old Score m (sd)	13-17-Year-Old Score m (sd)	Sig Testing^1^
*Scales*			
Anxiety	(*n* = 43) 8.46 (15.42)	(*n* = 35) 7.97 (8.78)	t(68.65) = 0.178, *p* = .860
Depression	(*n* = 43) 8.30 (13.69)	(*n* = 35) 6.17 (9.34)	t(73.92) = 0.814, *p* = .418
Anger	(*n* = 43) 8.02 (13.80)	(*n* = 35) 4.51 (6.55)	U = 644.000, *p* = .275
Post-Traumatic Stress	(*n* = 43) 10.67 (13.60)	(*n* = 35) 7.51 (8.16)	t(70.39) = 1.27, *p* = .209
*Dissociation*			
Dissociation	(*n* = 43) 5.02 (13.35)	(*n* = 35) 4.77 (8.00)	t(72.355) = 0.142, *p* = .887
Dissociation – Overt	(*n* = 43) 4.86 (13.57)	(*n* = 35) 5.31 (8.62)	t(73.98) = − 0.135, *p* = .893
Dissociation – Fantasy	(*n* = 44) 2.28 (11.18)	(*n* = 35) 2.63 (9.74)	t(77) = − 0.121, *p* = .904
*Sexual*			
Sexual Concerns	(*n* = 21) 6.19 (14.86)	(*n* = 24) 3.96 (11.40)	U = 254.500, *p* = .859
Sexual Concerns – Pre-Occupation	(*n* = 21) 5.62 (9.22)	(*n* = 24) − 0.46 (12.78)	U = 192.000, *p* = .164
Sexual Concerns - Distress	(*n* = 21) 4.05 (20.19)	(*n* = 24) 8.67 (14.23)	U = 224.500, *p* = .382

^1^ <0.05 with a Bonferroni Adjusted Alpha to 0.005

### Therapy Funding

Cases with treatment funded by the state child protection authority experienced similar improvement across scales to Medicare funded cases (see Table 7), with no significant differences. Notably, Medicare funded places had worsening symptoms on the Sexual Concerns – Pre-Occupation scale (*m* = -5.14; *sd* = 13.42) and a very small change on Sexual Concerns (*m* 0.50; *sd* = 13.42), this may have been because these tended to be cases referred from the co-located multi-agency response which included recent disclosures of child sexual abuse.

**Table 7:** Comparison of Symptom Change Across Therapy Funding Source (*n* = 59)

	Child Protection Funded Score m (sd)	Medicare Funded Score m (sd)	Sig Testing^1^
*Scales*			
Anxiety	(*n* = 30) 8.27 (12.55)	(*n* = 29) 7.55 (13.25)	*U* = 406.500, *p* = .665
Depression	(*n* = 30) 5.47 (12.63)	(*n* = 29) 8.28 (12.38)	*t*(57) = − 0.86, *p* = .392
Anger	(*n* = 30) 5.60 (13.85)	(*n* = 29) 8.10 (9.71)	*U* = 323.500, *p* = .090
Post-Traumatic Stress	(*n* = 30) 8.47 (12.11)	(*n* = 29) 10.38 (11.28)	*t*(59) = − 0.63, *p* = .533
*Dissociation*			
Dissociation	(*n* = 30) 2.40 (11.29)	(*n* = 29) 5.07 (12.22)	*U* = 395.000, *p* = .544
Dissociation – Overt	(*n* = 30) 2.43 (11.67)	(*n* = 29) 5.48 (12.72)	*t*(57) = − 0.96, *p* = .341
Dissociation – Fantasy	(*n* = 30) 1.00 (9.57)	(*n* = 29) 1.03 (10.80)	*t*(57) = − 0.013, *p* = .990
*Sexual*			
Sexual Concerns	(*n* = 13) 6.69 (12.73)	(*n* = 14) 0.50 (13.05)	*U* = 75.500, *p* = .316
Sexual Concerns – Pre-Occupation	(*n* = 13) 5.38 (10.59)	(*n* = 14) -5.14 (13.42)	*U* = 54.000, *p* = .076
Sexual Concerns - Distress	(*n* = 13) 5.62 (15.77)	(*n* = 14) 8.14 (13.96)	*U* = 93.000, *p* = .856

^1^ <0.05 with a Bonferroni Adjusted Alpha to 0.005

### Care Status

No significant differences in the extent of improvements on any scales were identified between cases in some form of out-of-home care and those not (see Table 8). While not significant, the not in care group appeared to have larger improvements on the depression (*t*(77) = -2.29, *p* = .025), anger (*U* = 631.000, *p* = .012), post-traumatic stress scales (*t*(77) = -2.54, *p* = .013). However, the not-in-care group (*m* = − 0.23; *sd* = 12.24) on average did not improve on the sexual concerns – pre-occupation scale in contrast with the in-care group (*m* = 6.00; *sd* = 9.48).

**Table 8:** Comparison of Symptom Change across Care Status (*n* = 78)

	In Care of the CEO Score m (sd)	Not in Care Score m (sd)	Sig Testing^1^
*Scales*			
Anxiety	(*n* = 35) 6.17 (14.09)	(*n* = 43) 9.93 (11.54)	*t*(76) = -1.30, *p* = .199
Depression	(*n* = 35) 4.00 (12.89)	(*n* = 43) 10.07 (10.42)	*t*(76) = -2.30, *p* = .024
Anger	(*n* = 35) 4.63 (9.97)	(*n* = 43) 7.93 (12.06)	*U* = 615.500, *p* = .168
Post-Traumatic Stress	(*n* = 35) 5.71 (11.34)	(*n* = 43) 12.14 (10.96)	*t*(76) = -2.53, *p* = .013
*Dissociation*			
Dissociation	(*n* = 35) 2.88 (12.35)	(*n* = 43) 6.56 (10.02)	*U* = 607.000, *p* = .143
Dissociation – Overt	(*n* = 35) 2.57 (12.74)	(*n* = 43) 7.09 (10.19)	*U* = 591.000, *p* = .104
Dissociation – Fantasy	(*n* = 35) 2.17 (10.71)	(*n* = 43) 2.65 (10.44)	*t*(76) = − 0.20, *p* = .842
*Sexual*			
Sexual Concerns	(*n* = 19) 8.84 (12.17)	(*n* = 26) 2.19 (13.13)	*t*(44) = 1.76, *p* = .086
Sexual Concerns – Pre-Occupation	(*n* = 19) 5.95 (9.74)	(*n* = 26) − 0.23 (12.24)	*U* = 187.000, *p* = .159
Sexual Concerns - Distress	(*n* = 19) 8.42 (17.58)	(*n* = 26) 5.12 (17.16)	*U* = 235.500, *p* = .625

^1^ <0.05 with a Bonferroni Adjusted Alpha to 0.005

### Presence of Child Sexual Abuse

On all scales no differences were found between groups with and without the presence of child sexual abuse in the case history (see Table 9).

**Table 9:** Comparison of Symptom Change Across Presence of CSA (*n* = 75)

	CSA Score m (sd)	No CSA Score m (sd)	Sig Testing
*Scales*			
Anxiety	(*n* = 35) 7.74 (13.20)	(*n* = 40) 8.18 (12.39)	*t*(73) = − 0.15, *p* = .884
Depression	(*n* = 35) 7.31 (11.07)	(*n* = 40) 7.50 (11.81)	*t*(73) = − 0.07, *p* = .944
Anger	(*n* = 35) 5.86 (13.74)	(*n* = 40) 7.12 (7.50)	*U* = 593.000, *p* = .255
Post-Traumatic Stress	(*n* = 35) 10.17 (10.99)	(*n* = 40) 8.22 (11.40)	*t*(73) = − 0.75, *p* = .456
*Dissociation*			
Dissociation	(*n* = 35) 4.86 (10.30)	(*n* = 40) 5.00 (11.64)	*U* = 665.000, *p* = .710
Dissociation – Overt	(*n* = 35) 5.06 (10.78)	(*n* = 40) 5.12 (11.85)	*U* = 669.500, *p* = .877
Dissociation – Fantasy	(*n* = 35) 1.94 (10.36)	(*n* = 40) 2.88 (10.88)	*t*(73) = − 0.38, *p* = .706
*Sexual*			
Sexual Concerns	(*n* = 25) 6.00 (13.96)	(*n* = 19) 3.42 (12.23)	*U* = 234.000, *p* = .712
Sexual Concerns – Pre-Occupation	(*n* = 14) 1.44 (14.15)	(*n* = 19) 3.10 (7.22)	*U* = 222.500, *p* = .716
Sexual Concerns – Distress	(*n* = 25) 9.08 (16.67)	(*n* = 19) 3.26 (18.27)	*U* = 234.000, *p* = .702

^1^ <0.05 with a Bonferroni Adjusted Alpha to 0.005

## Discussion

This article examined the treatment outcomes for a cohort of children receiving treatment for trauma associated with child abuse and neglect in a community clinic providing an adapted version of TF-CBT with other treatment components added based on matching treatments to symptoms (Chan & Herbert, 2022). Multiple maltreatment and complex symptoms of trauma present considerable challenges for existing treatment models, which are primarily designed around single types of traumas and without the overlay of multiple other issues in the family. Most of the major symptoms of trauma appear to be effectively addressed through the delivery of the hybrid TF-CBT approach, although symptoms related to sexual concerns, and dissociation symptoms among younger children did not appear to be influenced by the treatment. This may have been as on average symptoms were lower on the Dissociation – Fantasy and Sexual Concerns – Pre-Occupation scales for the sample. No significant differences in treatment effect were found across categories the sample was compared on (gender, age, funding source, care status, presence of CSA, living situation at intake). Overall, the study suggests that supplementing TF-CBT with additional therapeutic approaches may be helpful for children with multiple maltreatments and complex symptoms, but for the service delivering this approach (TFS) additional components that are known to address dissociation and sexual concerns may be required.

Looking to similar community-based treatment studies utilising TF-CBT, this study found much larger improvements than Ruiz (2016) and Konanur et al. (2015), but similar changes on the PTS scale as Kolko et al. (2011). Ruiz (2016) included only a short treatment period (3-months) among a sample of sexually abused children receiving TF-CBT from a community clinic. T1 scores on the TSCC scales were much lower than in the current study, and the treatment effect was smaller on all scales, although significant in the context of this study. Konanur et al. (2015) had a similar baseline of symptoms on the PTS scale of the TSCC from a community-based intervention for trauma exposed school age children in Canada, with TF-CBT delivered by a Children’s Advocacy Centre. The researchers reported only a small reduction on the PTS scale between ‘pre-therapy’ and ‘post-therapy’ measurement points, which are equivalent to the measurement period in the current study (Konanur et al., 2015). Kolko et al. (2011) included children with backgrounds of physical abuse in an adapted version of TF-CBT called Alternatives for Families: A Cognitive Behavioral Therapy (AP-CBT). The current study had slightly higher pre-test means for each scale than Kolko et al. (2011), and similarly had slightly higher mean improvements on symptoms. These differences may be artefacts of the sample in this study, which included referrals from sexual abuse investigations, state funded sexual abuse services, out of home care, and self-referrals, or could be related to the additional treatment approaches implemented as part of this program.

While the included sample was fairly distinct due to the sampling procedure and the sources of referral for the program, the study reflects the diversity of cases that may be referred to community-based clinics and the challenges of clinical psychology services to deliver evidence-based treatments to this population. Having to respond to complex and diverse client populations is increasingly common, as services are expected to implement a ‘no wrong door’ approach to therapy and support services (Royal Commission into Institutional Responses to Child Sexual Abuse, 2017). And there is increasing recognition that the overwhelming population of children in need of trauma treatment have experienced multiple forms of maltreatment and have complex issues within their families and care arrangements (Shevlin et al., 2018).

### Limitations

As noted in the method section, the sample did not match the treatment population on several characteristics. The sample was not chosen randomly, rather these cases were selected based on having repeated measures of the same instrument over a continuous treatment period. This meant that the sampled cases disproportionately included some of the more severe cases (both in terms of background and baseline symptoms), which received additional attention from the treatment team and were more likely to have multiple psychometric instruments administered. This limits the ability to extrapolate the findings to the whole treatment population, which on average were less likely to be in care, have parental mental health as a concern and have a background including child sexual abuse.

This study drew on data retrospectively, while information could be obtained about the number and frequency of treatment sessions this did not extend to the measurement of treatment fidelity/quality. Chan and Herbert (2022) outlines the treatment approach, however there was no data available to provide assurance of fidelity to the model, or to analyse how fidelity to the model related to treatment outcomes. Future work would ideally link the process of symptom matching and the treatment formulations arrived at with the improvement of symptoms, with the potential to study the effects of different types of formulation among similar symptom profiles.

As observed in the discussion, the pattern of engagement with therapy was complex, with most children having multiple periods of treatment, many of which were not bookended with the administration of a psychometric instrument. While the study reports on the number of therapy sessions that occurred prior to the first measure, this did not capture the multiple periods of contact often with significant gaps between them. These multiple periods of engagement complicate the observation of the treatment effects, for example a child may have engaged with therapy which reduced their symptoms, then re-engaged 4 months later and received their first measure of a psychometric instrument. This complexity makes it difficult to draw conclusions based on the number of sessions or treatment length.

### Implications

This adapted approach to therapy appears to address the major symptoms of trauma (anxiety, depression, post-traumatic stress, anger, dissociation) among a sample of children predominately presenting with complex trauma responses and complex histories of abuse and neglect. This broadly appears to support the approach of adapting TF-CBT to treat this target group, although a randomised trial would be required to compare this approach against a standard TF-CBT approach. This would also require a more formalised version of the approach to be implemented (Chan & Herbert, 2022) with appropriate fidelity checking. Despite similarly high symptomatology on the sexual concerns and dissociation scales on the TSCC, there appeared to be limited or no improvement on *Dissociation – Fantasy*, *Sexual Concerns, Sexual Concerns – Pre-Occupation* scales. This may suggest that the therapy team should explore additional therapies to add into the matrix that more directly address these symptoms.

For other treatment providers responding to similarly diverse and complex populations of children, there appears to be value in a symptom matching approach. Chan and Herbert (2022) covers some of the challenges experienced by the service provider in implementing this approach, namely maintaining training and competency across the clinician team in multiple approaches, managing individual clinician preferences as part of the formulation process, and the intensity of supervision and support needed.

As identified, the case histories of the sample were highly complex, often with multiple engagements and disengagements with therapy. The reasons for disengaging with therapy are complex and there is some evidence to suggest that barriers to engaging may vary considerably between service systems (Herbert, 2021). Further exploration of the patterns of disengagement and their relationship to the characteristics of children/young people may help to design a more effective system of referral and intake that addresses barriers to access (e.g., Budde et al., 2023).

## Footnotes

1 While other factors such as relationship to perpetrator, care history, caregiver support etc. are important factors relevant to the treatment of trauma, these were not routinely recorded or available in the collected data.

2 Parenting skills, relaxation skills, affect modulation skills, and cognitive processing skills.

## References

[CR1] Bisson, J. I., Berliner, L., Cloitre, M., Forbes, D., Jensen, T. K., Lewis, C., Monson, C. M., Olff, M., Pilling, S., Riggs, D. S., Roberts, N. P., & Shapiro, F. (2019). The International Society for Traumatic Stress Studies New Guidelines for the Prevention and Treatment of PTSD: Methodology and development process. *Journal of Traumatic Stress*, *32*, 475–483. 10.1002/jts.22421. 31283056 10.1002/jts.22421

[CR2] Briere, J. (1996). *Trauma Symptom Checklist for children: Professional Manual*. Psychological Assessment Resources, Inc.

[CR3] Briere, J. (2005). *Trauma symptom checklist for young children (TSCYC)*. Psychological Assessment Resources.

[CR4] Budde, S., Walsh, W., Waters, J., Kacha-Ochana, A., & Irving, K. (2023). A children’s Advocacy Centre Comprehensive Initiative to increase Engagement with Children’s Mental Health Services. In A. St-Amand, D. Nadeau, P. Rimer, J. Herbert, & W. Walsh (Eds.), *Contemporary and Innovative Practices in Child & Youth Advocacy Centre Models* (pp. 253–271). University of Quebec.

[CR5] Celano, M., NeMoyer, Amanda, Stagg, A., & Scott, N. (2018). Predictors of treatment completion for families referred to trauma-focused cognitive behavioral therapy after child abuse. *Journal of Traumatic Stress*, *31*(3), 454–459. 10.1002/jts.22287. 29786886 10.1002/jts.22287

[CR6] Chan, S., & Herbert, J. (2022). Parkerville Children and Youth Care Inc. therapeutic treatment model practice mapping. University of South Australia. https://www.unisa.edu.au/siteassets/research/accp/230220-practice-mapping-report-.pdf.

[CR7] Cohen, J. A., & Mannarino, A. P. (1996). A treatment outcome study for sexually abused preschool children: Initial findings. *Journal of the American Academy of Child & Adolescent Psychiatry*, *35*(1), 42–50. 10.1097/00004583-199601000-00011. 8567611 10.1097/00004583-199601000-00011

[CR8] Cohen, J. A., & Mannarino, A. P. (2000). Predictors of treatment outcome in sexually abused children. *Child Abuse & Neglect*, *24*(7), 983–994. 10.1016/S0145-2134(00)00153-8. 10905421 10.1016/s0145-2134(00)00153-8

[CR9] Cohen, J. A., Mannarino, A. P., Kliethermes, M., & Murray, L. A. (2012). Trauma-focused CBT for youth with complex trauma. *Child Abuse & Neglect*, *36*(6), 528–541. 10.1016/j.chiabu.2012.03.007. 22749612 10.1016/j.chiabu.2012.03.007 PMC3721141

[CR10] Cook, A., Spinazzola, J., Ford, J., Lanktree, C., Blaustein, M., Cloitre, M., DeRosa, R., Hubbard, R., Kagan, R., Liautaud, J., Mallah, K., Olafson, E., & van der Kolk, B. (2005). Complex trauma in children and adolescents. *Psychiatric Annals*, *35*(5), 390–398. 10.3928/00485713-20050501-05.

[CR11] DeLorenzi, L., Daire, A. P., & Bloom, Z. D. (2016). Predicting treatment attrition for child sexual abuse victims: The role of child trauma and co-occurring caregiver intimate partner violence. *Counseling Outcome Research and Evaluation*, *7*(1), 40–52. 10.1177/2150137816632850.

[CR12] Hébert, M., & Amédée, L. M. (2020). Latent class analysis of post-traumatic stress symptoms and complex PTSD in child victims of sexual abuse and their response to trauma-focused cognitive behavioural therapy. *European Journal of Psychotraumatology*, *11*(1), 1807171. 10.1080/20008198.2020.1807171. 33062212 10.1080/20008198.2020.1807171 PMC7534355

[CR13] Herbert, J. L. (2021). Rates of therapy use following a disclosure of child sexual abuse. *Child Family Community Australia, 58*. https://aifs.gov.au/sites/default/files/publication-documents/2105_cfca_58_rates_of_therapy_utilisation_0_0.pdf.

[CR14] Herbert, J. L., & Bromfield, L. (2020). Worker perceptions of the Multi-agency Investigation & Support Team (MIST): A process evaluation of a cross-agency response to severe child abuse. *Journal of Child Sexual Abuse*, *29*(6), 638–658. 10.1080/10538712.2019.1709241. 32045339 10.1080/10538712.2019.1709241

[CR15] Herbert, J. L., & Bromfield, L. M. (2021). A quasi-experimental study of the Multi-agency Investigation & Support Team (MIST): A collaborative response to child sexual abuse. *Child Abuse & Neglect*, *111*, 104827. 10.1016/j.chiabu.2020.104827. 33250277 10.1016/j.chiabu.2020.104827

[CR16] Higgins, D. J., Mathews, B., Pacella, R., Scott, J. G., Finkelhor, D., Meinck, F., Erskine, H. E., Thomas, H. J., Lawrence, D. M., Haslam, D. M., Malacova, E., & Dunne, M. P. (2023). The prevalence and nature of multi-type child maltreatment in Australia. *The Medical Journal of Australia*, *218*(S6), S19–S25. 10.5694/mja2.51868. 37004183 10.5694/mja2.51868 PMC10952595

[CR17] Jensen, T. K., Holt, T., Ormhaug, S. M., Egeland, K., Granly, L., Hoaas, L. C., Hukkelberg, S. S., Indregard, T., Stormyren, S. D., & Wentzel-Larsen, T. (2014). A randomized effectiveness study comparing trauma-focused cognitive behavioral therapy with therapy as Usual for youth. *Journal of Clinical Child & Adolescent Psychology*, *43*(3), 356–369. 10.1080/15374416.2013.822307. 23931093 10.1080/15374416.2013.822307 PMC4037845

[CR18] Kisiel, C., Fehrenbach, T., Small, L., & Lyons, J. S. (2009). Assessment of complex trauma exposure, responses, and service needs among children and adolescents in child welfare. *Journal of Child & Adolescent Trauma*, *2*(3), 143–160. 10.1080/19361520903120467.

[CR19] Kliethermes, M., Schacht, M., & Drewry, K. (2014). Complex trauma. Child and adolescent. *Psychiatric Clinics of North America*, *23*, 339–361. 10.1016/j.chc.2013.12.009. 10.1016/j.chc.2013.12.009 24656584

[CR20] Kolko, D. J., Iselin, A. M. R., & Gully, K. J. (2011). Evaluation of the sustainability and clinical outcome of Alternatives for families: A cognitive-behavioral therapy (AF-CBT) in a child protection center. *Child Abuse & Neglect*, *35*(2), 105–116. 10.1016/j.chiabu.2010.09.004. 21354619 10.1016/j.chiabu.2010.09.004 PMC3069689

[CR21] Konanur, S., Muller, R. T., Cinamon, J. S., Thornback, K., & Zorzella, K. P. (2015). Effectiveness of trauma-focused cognitive behavioral therapy in a community-based program. *Child Abuse & Neglect*, *50*, 159–170. 10.1016/j.chiabu.2015.07.013. 26318778 10.1016/j.chiabu.2015.07.013

[CR22] Maercker, A., Cloitre, M., Bachem, R., Schlumpf, Y. R., Khoury, B., Hitchcock, C., & Bohus, M. (2022). Complex post-traumatic stress disorder. *The Lancet*, *400*(10345), 60–72. 10.1016/S0140-6736(22)00821-2. 10.1016/S0140-6736(22)00821-2 35780794

[CR23] Royal Commission into Institutional Responses to Child Sexual Abuse (2017). Final Report: Advocacy, Support and Therapeutic Treatment Services (Volume 9). Commonwealth of Australia. https://www.childabuseroyalcommission.gov.au/sites/default/files/final_report_-_volume_9_advocacy_support_and_therapeutic_treatment_services.pdf.

[CR24] Ruiz, E. (2016). Trauma symptoms in a diverse population of sexually abused children. *Psychological Trauma: Theory Research Practice and Policy*, *8*(6), 680. 10.1037/tra0000160. 27243569 10.1037/tra0000160

[CR25] Self-Brown, S., Tiwari, Ashwini, Lai, Betty, Roby, S., & Kinnish, K. (2016). Impact of caregiver factors on Youth Service utilization of trauma-focused cognitive behavioral therapy in a community setting. *Journal of Child and Family Studies*, *25*(6), 1871–1879. 10.1007/s10826-015-0354-9.

[CR26] Shevlin, M., Murphy, S., Elklit, A., Murphy, J., & Hyland, P. (2018). Typologies of child sexual abuse: An analysis of multiple abuse acts among a large sample of Danish treatment-seeking survivors of childhood sexual abuse. *Psychological Trauma: Theory Research Practice and Policy*, *10*(3), 263. 10.1037/tra0000268. 28368155 10.1037/tra0000268

[CR27] Stanaway, J. D., Afshin, A., Gakidou, E., Lim, S. S., Abate, D., Abate, K. H., et al. (2018). Global, regional, and national comparative risk assessment of 84 behavioural, environmental and occupational, and metabolic risks or clusters of risks for 195 countries and territories, 1990–2017: A systematic analysis for the global burden of Disease Study 2017. *The Lancet*, *392*(10159), 1923–1994. 10.1016/S0140-6736(18)32225-6. 10.1016/S0140-6736(18)32225-6 PMC6227755 30496105

[CR28] Wolfe, D. A., Francis, K. J., & Straatman, A. L. (2006). Child abuse in religiously-affiliated institutions: Long-term impact on men’s mental health. *Child Abuse & Neglect*, *30*(2), 25–212. 10.1016/j.chiabu.2005.08.015. 10.1016/j.chiabu.2005.08.015 16464495

[CR29] World Health Organization (2018). International statistical classification of diseases and related health problems (11th review). https://www.who.int/classifications/icd/en. 10.2471/BLT.25.294650 PMC13531092 42683092

